# Physical Therapy in Tension-Type Headache: A Systematic Review of Randomized Controlled Trials

**DOI:** 10.3390/ijerph20054466

**Published:** 2023-03-02

**Authors:** Angela Repiso-Guardeño, Noelia Moreno-Morales, María Angeles Armenta-Pendón, María del Carmen Rodríguez-Martínez, Ricardo Pino-Lozano, Juan Antonio Armenta-Peinado

**Affiliations:** 1 Department of Physiotherapy, Faculty of Health Sciences, University of Málaga, C/Arquitecto Francisco Peñalosa, 3, 29071 Málaga, Spain; 2 Clínica de Fisioterapia Ángela Repiso, Villanueva del Rosario, 29312 Málaga, Spain; 3 Instituto de Investigación Biomédica de Málaga y Plataforma en Nanomedicina—IBIMA Plataforma BIONAND (IBIMA Plataforma BIONAND), 29590 Málaga, Spain; 4 Clínica Bonal—Centro Médico de Especialidades, Torre del Mar, 29740 Málaga, Spain; 5 Centre d´Atenció Primària Vilafranca Nord, Institut Catalá de la Salut, Vilafranca del Penedès, 08720 Barcelona, Spain

**Keywords:** tension-type headache, physical therapy modalities, physical therapy interventions

## Abstract

(1) Objective: The aim of this study is to synthesize the effects of physical therapy on pain, frequency, or duration management in the short, medium, and long term in adult patients diagnosed with Tension-type headache (TTH). (2) Background: Tension-type headache (TTH) is the most common headache with migraine and its pathophysiology and treatment has been discussed for years without reaching a consensus. (3) Methods: A systematic review was conducted using the Preferred Reporting Items for Systematic Reviews and Meta-Analyses (PRISMA) guidelines. The review was registered in PROSPERO (CRD42020175020). The systematic search for clinical trials was performed in the databases PubMed, CINAHL, Cochrane Central Register of Controlled Trials, PEDro, Scopus, SciELO and Dialnet. Articles were selected according to the inclusion and exclusion criteria, regarding the effectiveness of physical therapy interventions on adult patients with TTH published in the last 11 years with a score ≥ 6 in the PEDro Scale (Physiotherapy Evidence Database). (4) Results: In total, 120 articles were identified, of which 15 randomized controlled trials were finally included in order to determine the inclusion criteria. Changes in pain intensity, headache frequency or headache duration of individual studies were described (5) Conclusions: This systematic review shows that there is no standardized physical therapy protocol for the approach to tension headache, although all the techniques studied to date address in one way or another the cranio-cervical-mandibular region. The approach to the cranio-cervical-mandibular region reports significant effects in terms of decreasing the intensity of pain and frequency of headache episodes in the short and medium term. More long-term longitudinal studies are needed.

## 1. Introduction

The most frequent type of headache, tension-type headache (TTH), is addressed frequently but there is no agreement on its pathogenesis, making adequate care difficult for medical professionals [1]. According to the Global Burden of Disease (GBD, 2019), headaches are among the most prevalent conditions worldwide, with tension-type headaches (TTH) estimated worldwide at an average of 26.0% (22.7–29.5%), with 23.4% in men and 27.1% in women [2]. Geographically, the prevalence of TTH ranged from 11.1% (Southeast and East Asia and Oceania) to 33.1% in South Asia. However, when adjusting the population residing geographically in the different regions, it is found that the prevalence of TTH was 21.1% [2].

By age, between 20 and 64 years, TTH is the most prevalent of all headaches. The profile of a patient diagnosed with tension headache is female (80%), white (65%) and with an average age of 40 years [1]. The International Classification of Headache Disorders (ICHD) of 2018 distinguishes three subtypes of Tension-type Headache (TTH), according to the frequency of the episodes: Infrequent Episodic Tension-Headache type (IETTH), Frequent Episodic Tension-type Headache (FETTH) and Chronic Tension-type Headache (CTTH) [3].

Despite this, all subtypes of tension-type headaches share a common characteristic: bilateral non-pulsatile pain of oppressive nature and mild to moderate intensity does not worsen with movement or is associated with nausea or vomiting. Mild nausea exclusively appears in the Chronic Tension-type Headache [3].

In addition, the patient who suffers from it may have discomfort when exposed to light or noise, but not both at the same time and bears at least 10 episodes per year witha duration ranging from 30 min to 7 days [3]. Regarding its location, the pain is focused on the parietal, frontal and suboccipital region of the head [4].

The majority of migraine patients suffer episodes where it is combined with TTH and do not know it. The diagnosis of TTH is exclusively clinical because there are no biological indicators associated with this type of headache. Therefore, diary headaches, a detailed history and clinical examinations which rule out other associated pathologies are essential for its diagnosis [5].

Regarding its etiology, the cranial and neck musculature, stress, and central sensitization are among the potential culprits [6]. It is likely that peripheral myofascial nociceptors are involved in the activation or sensitization of myofascial nociceptors, which is involved in the development of muscle pain and the acute episode of TTH. Repetitive episodes of muscle pain can sensitize the central nervous system, causing TTH to progress. Therefore, muscle variables may be to blame for both the chronic nature of the disease as well as the episode of acute headache [6].Tension-type Headache, although a priori is associated with mild symptoms, can become disabling when the symptomatology is aggravated and may increase work absenteeism, decrease social commitments and in some cases, may trigger depressive states in the person suffering from it [7].Even stress and anxiety are triggering factors that are having a lot of significance in the development of TTH [8].In this sense, a recent study by Safiri et al. [9], highlighting that the increase in theincidence of TTH in the Middle East and North Africa region could be related to an increase in psychiatric problems, being a 30% higher incidence of anxiety, stress and depression, compared to the world average. In another recent study, the average TTH in this region was 20.5% [2].

Other studies found gender differences in the presence of Trigger Points (TrPs) in patients with TTH, especially in the temporal, suboccipital and splenius capitis muscles. Women with TTH exhibited a greater number of active TrPs, especially in temporal and suboccipital muscles, and a pressure threshold lower than men [10].

Episodic TTH can evolve into the chronic form due to different aspects and several triggers may be involved at the same time. Components such as muscle stress, lack of relaxation and factors such as posture, sleep disturbances and medication abuse cause symptoms to be triggered more frequently and even TTH to become chronic [11].

Currently, the most accepted model to explain the origin of pain in patients with chronic tension headache is based on sensitization [10]. According to this model, peripheral nociception could come from active myofascial trigger points, the muscles that are innervated by the upper cervical segments C1–C3 with active myofascial trigger points (PGMs) (upper fasciculus of the trapezius muscle, suboccipital muscles, sternocleidomastoid muscle) and by the trigeminal nerve (temporal muscle, masseter muscle). If this nociception were prolonged over time, it would represent a continuous afferent bombardment of the trigemini-cervical nucleus, a situation that would sensitize the central nervous system. In addition, the presence of active PGMs in suboccipital, upper trapezius, temporal, sternocleidomastoid and extraocular muscles have been associated in several studies with headaches of greater intensity, frequency and duration as well as with a greater hypersensitivity to pressure on them [10].

At the European level, tension headache is a very high economic cost. This is due to the consumption of drugs, the performance of diagnostic tests and visits to the Emergency Room. To all the above, the high indirect cost represented by the days of sick leave and the decrease in performance at work [3].

Among the therapeutic approaches for TTH is Physiotherapy [6], since it can help reduce the intensity and frequency of headaches, improving mobility and functionality, which will result in an improvement in the quality of life of these patients.

Non-pharmacological treatments may include counseling (postural and ergonomic education), biofeedback, manipulative therapy, muscle relaxation training, massage, therapeutic exercise and acupuncture, as part of the physiotherapy care process to help reduce TTH symptoms. In order to achieve a positive effect with these techniques, once the origin of the problem has been correctly diagnosed, an adequate and personalized muscular, postural and biomechanical evaluation must be initiated, which will allow the choice of the most appropriate therapeutic procedure for the patient’s condition [4].

The aim of this study is to synthesize the effects of physical therapy on pain, frequency, or duration management in the short, medium, and long term in adult patients diagnosed with tension-type headache.

## 2. Materials and Methods

### 2.1. Eligibility Criteria

The purpose of this study is to find and synthesize the results of the RCTs published in the last 11 years with a score of ≥6 in the PEDro Scale examining the effectiveness of physical techniques on adult patients with Tension-Type Headache (TTH).

Our research question was established following recommendations from the PICO model (Population, Intervention, Comparison and Outcome measures). Patients included were both male and female adult subjects, clinically diagnosed of TTH. Intervention was any type of physical therapy modality compared with another intervention group, control or placebo. Outcome measures were pain intensity, headache frequency and headache duration.

### 2.2. Information Sources and Search Strategy

This systematic review of Randomized Controlled Trials (RCTs) was performed following PRISMA guidelines (Preferred Reporting Items for Systematic Reviews and Meta-Analyses) [12]. An electronic search was conducted in January 2022 on the following databases: PubMed, CINAHL, Cochrane Central Register of Controlled Trials (CENTRAL), PEDro, SCOPUS, SciELO and Dialnet. Strategy search was a combination of the following MeSH-listed key words: ((physical therapy modalities) OR (physical therapy interventions)) AND (Tension-type headache). The restriction of the year of publication was 11 years. RCTs in Spanish and English language were included.

### 2.3. Study Selection

The inclusion criteria were:

- Study design. Randomized controlled trials;
- Population: Adults (19+ years) diagnosed with TTH;
- Intervention: Physical therapy. The intervention must be led by a physiotherapist and the procedure must be within their competence;
- Score ≥ 6/10 in the PEDro scale;
- Comparison: placebo, active intervention or no therapy.
- Language: English and Spanish

The exclusion criteria were:

- Studies with different style than RCT’s (reviews, cohort studies, ongoing ones or pilot, etc.);
- Not evaluating the effectiveness of physical therapy on intensity of pain or frequency of headache;
- Sample of patients with other types of headaches such as migraine or as side effect of another disease;
- Articles which were published prior to January 2011.

### 2.4. Data Collection Process

Articles were selected by screening title and abstract, and duplicates were removed. After that, the analysis of selected full text studies was performed. Inclusion and exclusion criteria were then checked by two independent reviewers. When articles for this research were identified, quality assessment (risk of bias) was conducted independently by three investigators. Any disagreement on quality assessment was resolved by consensus.

The data extraction process extracted the following information from each study: first author, publication year, sample size, population main characteristics, treatment methods and duration, comparison group characteristics, measurement tools and follow up period. Main outcomes were pain intensity and headache frequency. The secondary outcome was headache duration.

### 2.5. Risk of Bias in Individual Studies and Summary Measures

The methodological quality of the RCTs was assessed according to the PEDro scale (Physiotherapy Evidence Database) [13]. Although the scale is composed of 10 questions with YES/NO answers, only questions regarding internal validity were used. Accordingly, a clinical trial evaluated with the PEDro scale which presents 6 or more affirmative responses is considered level I (6–8: good and 8–10: excellent) and a clinical trial with a score equal to or less than 5 is considered level II (4–5: fair and <4: poor).

Main outcomes were pain intensity and headache frequency. The secondary outcome was headache duration. Results for primary and secondary outcome measures were thoroughly described. *p* value > 0.05 was considered statistically significant.

## 3. Results

### 3.1. Study Selection

The database search in the cited databases produced a total of 120 articles identified as potentially eligible, 20 of which were excluded as duplicates. A screening of the 100 remaining articles was performed based on titles and abstracts. 10 studies were rejected for not belonging to the field of physical therapy, resulting in 90 articles potentially eligible that were excluded following the exclusion criteria: not RCT studies (reviews, cohort studies, ongoing ones or pilot, proceeding, etc.) [10,14,15,16,17,18,19,20,21,22,23,24,25,26,27,28,29,30,31,32,33,34,35,36,37,38,39,40,41,42,43,44,45,46,47,48,49,50], sample of patients with other types of headaches such as migraine or as side effect of another disease [51,52,53,54,55,56,57,58,59,60,61,62,63,64,65,66,67], articles published prior to January 2011 [68] and studies that not evaluating the effectiveness of physical therapy on intensity of pain, frequency of headache or duration of the episodes [27,69,70,71,72,73,74,75,76,77].

**Figure 1:**
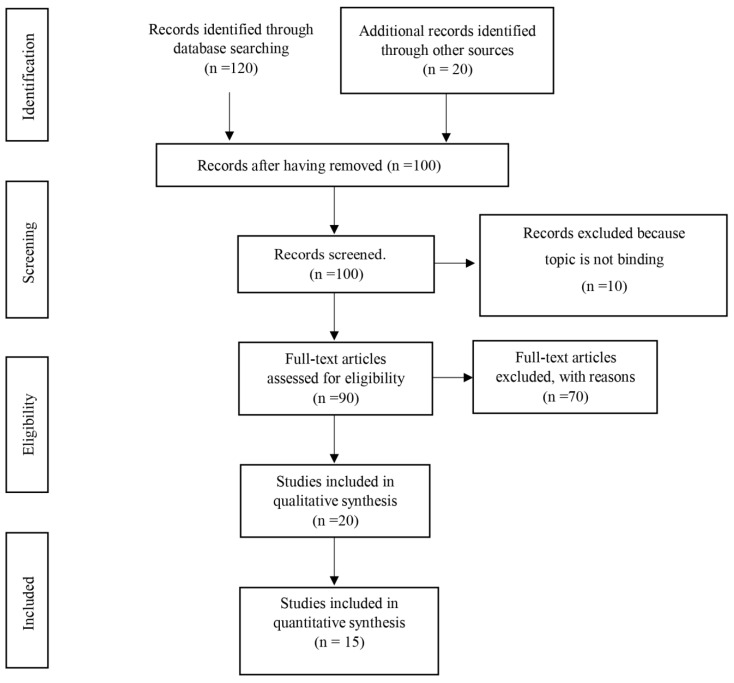
PRISMA 2020 flow diagram.

Likewise, having a score < 6/10 in the PEDro scale [78,79,80,81,82] implied an exclusion for this manuscript. Finally, 15 articles were included in this systematic review of randomized controlled trials [83,84,85,86,87,88,89,90,91,92,93,94,95,96,97]. The process of selection of studies is reflected in Figure 1.

### 3.2. Study Characteristics and Risk of Bias within Studies

Of the 15 RCTs included in the present review, 33% of them obtained a PEDro Scale score of 6/10, 47% a score of 7/10, 13% a score of 8/10 and 7% a score of 9/10. In all of them, outcome measures were obtained for at least one of the study variables (intensity, frequency or duration of headache). These data are grouped in Table 1.

The characteristics of all the studies included in this review are set out in Table 2.

### 3.3. Results of Individual Studies

Castien et al. [83] reported in their study that the techniques of mobilization of the cervical and thoracic spine accompanied by a postural re-education program of the head and neck significantly improved in the medium term, 8 weeks after intervention, the intensity (*p* = 0.003) and the frequency of chronic tension headache (*p* < 0.001). The techniques do notoverlaps with the others.

They also improved significantly in the long term, 26 weeks post-intervention, pain intensity (*p* = 0.027) and frequency of episodes (*p* < 0.001). The duration of headache episodes was significantly reduced in the medium term, 8 weeks post-intervention (*p* = 0.013). Ajimsha [84] concluded in his study that both direct myofascial release techniques and indirect myofascial release techniques significantly decrease the frequency of tension headache episodes in the medium term, 3 months post-intervention (*p* < 0.001).

Da Silva et al. [85] showed that traditional acupuncture and splenium trigger points of the neck, masseter and temporal decreases the intensity of tension headache in pregnant women in the medium term, 3 months post intervention (*p* = 0.035).

Berggreen et al. [86] demonstrated that the treatment of trigger points of the cranio-cervical-mandibular musculature significantly decreases the morning intensity of headache in patients with chronic tension headache in the short term, post-intervention period (*p* = 0.047).

Espí-López et al. [87] concluded that suboccipital inhibition decreases the intensity of tension headache in the short term, after the intervention period (*p* = 0.04). In addition, the manipulation of the upper cervical vertebrae decreases in the short term, after the intervention period, the intensity (*p* = 0.004) and the frequency of episodes of tension headache (*p* = 0.03). Finally, when the intervention combines suboccipital inhibition and manipulation of the upper cervical vertebrae, tension headache (*p* = 0.01) and the frequency of episodes (*p* = 0.02) also decrease significantly in the short term, after the intervention period.

Moraska et al. [88] showed that massage therapy and treatment of trigger points significantly decreases the frequency of headache episodes in the short term, 1 month after the intervention, (*p* = 0.026).

Chassot et al. [89] showed that electroacupuncture significantly decreases the intensity of tension headache in the short term, after the intervention period, (*p* = 0.005).

Espí-López et al. [90] concluded that manipulation of upper cervical spine combined with head and neck massage therapy decreases the frequency of headache episodes in the medium term, 2 months post-intervention (*p* < 0.01).

Ferragut-Garcías et al. [91] concluded that craniocervical soft tissue techniques, craniocervical neural mobilization techniques and combined treatment of both decrease the intensity (*p* < 0.001) and frequency (*p* < 0.001) of short-term, post-intervention period and 1-month post-intervention tension headache episodes.

Georgoudis et al. [92] concluded that microwave waves combined with myofascial treatment of the cranio-cervical-mandibular region decrease the intensity of tension headache in the short term, post-intervention period (*p* < 0.05).

Pérez-Llanes et al. [93] concluded that the combined treatment of suboccipital inhibition and interference currents does not significantly reduce the intensity of chronic tension headache in the short term, 1-month post-intervention (*p* = 0.18)

Schiller et al. [94] revealed that patients who received a combined treatment of acupuncture and medical training reduced in the medium term, 3 months after intervention, their mean pain intensity compared to the control group (*p* = 0.012), as well as the values of maximum intensity (*p* = 0.014) and minimum (*p* = 0.03). The frequency of pain (days/month) did not reflect significant differences between the different groups. In all groups, the duration of painful episodes and medication was reduced, showing a response rate of more than 90%. They concluded that tension headaches with pericranial sensitivity responded better to separate medical training, and those without sensitivity to treatment with acupuncture or combined with medical training.

Corum et al. [95] concluded that cervical manipulation significantly decreases the frequency of tension headache in the short, post intervention period (*p* < 0.05) and in the medium term, 3 months after intervention (*p* < 0.05) as well as the intensity of tension headache in the short term, post intervention period (*p* < 0.05) and medium term, 3 months after intervention (*p* < 0.05). They also concluded in their study that suboccipital inhibition significantly decreases the frequency of tension headache in the short term, post-intervention period, (*p* < 0.05) and in the medium term, 3 months post-intervention (*p* < 0.05). Finally, suboccipital inhibition decreases the intensity of tension headache in the short term, after the intervention period (*p* < 0.05) and in the medium term, 3 months after intervention (*p* < 0.05).

Gopichandran et al. [96] concluded that progressive relaxation of the jaw and neck muscles combined with deep breathing exercises decrease the intensity of chronic tension headache in the short term, 4 post intervention (*p* < 0.001) and medium term, 8 weeks and 3 months post intervention (*p* < 0.001).

Runa et al. [97] concluded that the combined treatment of moxibustion at the temples and the taking of the drug Deanxit decrease the intensity (*p* < 0.05), frequency (*p* < 0.05) and duration (*p* < 0.5) of tension headache in patients with anxiety in the short term, post intervention period. The summary of individual results of each study in this review are set out in Table 3.

## 4. Discussion

This systematic review focuses on analyzing the effectiveness of physical therapy in adult patients diagnosed with Tension-type headache (TTH). The guiding line when showing the results of the articles, follows an axis of effectiveness in time (short, medium or long term). In the short term: direct treatment of trigger points [86,88], suboccipital inhibition and manipulation of the upper cervical vertebrae (applied singly or together) [87,95], electroacupuncture [89], techniques of craniocervical soft tissue and upper craniocervical neural mobilization techniques (applied singly or together) [91], microwave waves combined with myofascial therapy [92], progressive relaxation of the jaw and neck muscles combined with deep breathing exercises [96] and moxibustion on the temples. Without short-term effect, the combined treatment of suboccipital inhibition and interferential currents is also found [93].

In the medium term, between 8 weeks and 3 months after treatment, there are mobilization techniques accompanied by a postural re-education program [83], direct and indirect myofascial release techniques [84], traditional acupuncture together with treatment of trigger points in the face and neck or with medical training [85,94], manipulation of the upper cervical spine alone or in combination with head and neck massage [90,95], suboccipital inhibition [95] and progressive relaxation of the jaw and neck muscles combined with deep breathing exercises [96].

In the long term, beyond 36 weeks of intervention, only the mobilization techniques accompanied by a postural re-education program applied by Castien et al. [38].

Most of the studies included in this review have reported results for the variables ‘headache intensity’ and ‘frequency of headache episodes’ [83,87,88,90,91,94,95,97], a lower percentage only for the variable ‘headache intensity’ [85,86,89,92,93,96] and one study only for the variable ‘frequency of headache episodes’ [84].

Four of the studies included in this systematic review have also reported results for the variable “duration of headache episodes” [83,86,94,97] and only one reported significant differences for this variable [83].

In the last eleven years, different protocols of action have been studied, such as mobilization of the cervical and thoracic spine accompanied by a program of postural re-education of the head and neck [83], myofascial release techniques [84], traditional acupuncture and in splenium trigger points of the neck, masseter and temporary [85], treatment of head trigger points, neck and jaw [86], suboccipital inhibition and manipulation of the upper cervical vertebrae, isolated and combined [87], massage therapy and treatment of trigger points in the cranio-cervical-mandibular region [88], general electroacupuncture [89], manipulation of upper cervical vertebrae [90], neural mobilization techniques and relaxation of the cranio-cervical soft tissue [91], microwave and myofascial treatment of the cranio-cervical-mandibular region [92], suboccipital inhibition combined with interference currents in lower cervical spine [93], acupuncture, training and combined treatment of both [94], cervical manipulation combined with suboccipital inhibition [95], progressive relaxation of the jaw and neck muscles combined with deep breathing exercises [96] or moxibustion in temples combined with taking the drug Deanxit [97].

Although the physical therapy protocols in the treatment of adult patients with tension headache are very varied. All of them, except the general electroacupuncture [86], address in one way or another, neck, head and/or mouth.

General electroacupuncture has only been reported to significantly reduce the intensity of tension headache in the short term [89]. General acupuncture and trigger points acupuncture on cranio-cervical-mandibular musculature, such as splenium, masseter and temporal muscles, has been studied in the medium term, three months after treatment, reporting significant improvement in the intensity of tension headache in pregnant women [85]. Acupuncture combined with a training program has also been shown to significantly improve the intensity of chronic headache in the medium term, three months post-intervention [94].

The manipulation of upper cervicalspine did not report significant differences in the short term either for the intensity of pain or for the frequency of headache episodes when compared with massage of the cranio-cervical region [90]. Nevertheless, it did report significative intragroup differences in pre-post treatment results when isolated [87].

Only the mobilization of the cervical and thoracic spine accompanied by a program of postural re-education of the head and neck in patients with chronic tension headache has reported significant long-term results, 26 weeks post-intervention, for frequency and intensity of headache episodes variables [83]. In this study, the duration of headache episodes did not decrease significantly in the long term, but it did in the medium term, eight weeks post-treatment.

In general, all studies reported significant improvement in some of the study variables.

A recent study related to our systematic review of Krøll et al. [98], conclude that non-pharmacological treatment approaches for TTH, used as an adjunct, are safe, free of significant adverse effects and with positive effects, although with low or very low evidence, especially [98]:

- Acupuncture could have positive effects in terms of pain intensity and frequency.
- Supervized physical activity could have a positive effect on pain intensity at the end of treatment and frequency during follow-up, although the recommendation for use is weak.
- Manual joint mobilization techniques (myofascial release and manipulation of the suboccipital muscles) could have a positive effect on frequency and quality of life during follow-up.

The evidence after the analysis of the results is very low, in fact the authors justify that solid conclusion cannot be drawn from this review [98]. However, in our review, one of the inclusion criteria for the analysis of the RCTs was that they had a score equal to or greater than 6 on the PEDro scale, so the methodological quality is higher and the conclusions obtained are more solid.

## 5. Conclusions

This systematic review found that, to date, there is no consensus on a single physical therapy protocol in the treatment of adult patients diagnosed with tension headache.

The techniques used to address this pathology are very diverse. So, there is no standardized physical therapy protocol for tension-type headache treatment, although all the techniques studied to date address in one way or another the cranio-cervical-mandibular region. The approach of the cranio-cervical-mandibular region reports significant effects in terms of decreasing the intensity of pain and frequency of headache episodes.

The studies included in this systematic review are heterogeneous in methodology and only one of them performed long term follow-ups on the intervention, 26 weeks post-intervention, for two study variables, intensity and frequency of pain: mobilization of the cervical and thoracic spine accompanied by a postural reeducation program of the head and neck.From these results, it would be interesting for future studies to analyze in depth etiological factors such as the relationship between the appearance of chronic tension headaches and the position of the head and neck..

Finally, this systematic review can serve as useful basis for promoting a consensus on non-pharmacological approaches to tension-type headache.

## 6. Limitations

The studies included in this systematic review are heterogeneous in methodology and only one of them performed long term follow-ups on the intervention, 26 weeks post-intervention.

The effectiveness of physical therapy with respect to the duration of headache episodes has not been analyzed by almost any of the studies. For that reason, more studies are needed regarding the effects of long-term physical therapy, as well as its effects on the duration of headache episodes in people with tension headache.

## Figures and Tables

**Table 1:** Risk of bias within studies using PEDro Scale.

Study	Criterion 1	Criterion 2	Criterion 3	Criterion 4	Criterion 5	Criterion 6	Criterion 7	Criterion 8	Criterion 9	Criterion 10	Criterion 11	Total Score
Castien et al. [83]		1	1	1	0	0	1	1	1	1	1	8/10
Ajimsha [84]		1	0	1	0	0	1	1	0	1	1	6/10
Da Silva et al. [85]		1	1	0	0	0	1	1	0	1	1	6/10
Berggreen et al. [86]		1	1	1	0	0	0	1	1	1	1	7/10
Espí-López et al. [87]		1	0	1	0	0	1	1	1	1	1	7/10
Moraska et al. [88]		1	1	1	0	0	0	1	0	1	1	6/10
Chassot et al. [89]		1	1	1	1	0	1	1	1	1	1	9/10
Espí-López et al. [90]		1	1	1	0	0	1	1	0	1	1	7/10
Ferragut-Garcías et al. [91]		1	1	1	0	0	1	1	0	1	1	7/10
Georgoudis et al. [92]		0	0	1	0	0	1	1	1	1	1	6/10
Pérez-Llanes et al. [93]		1	1	1	0	0	0	1	1	1	1	7/10
Schiller et al. [94]		1	1	1	0	0	0	1	1	1	1	7/10
Corum et al. [95]		1	1	1	0	0	1	1	1	1	1	8/10
Gopichandran et al. [96]		1	1	1	0	0	0	1	1	1	1	7/10
Runa et al. [97]		1	0	1	0	0	0	1	1	1	1	6/10
Espí-López et al. [78]		1	0	1	0	0	0	1	0	1	1	5/10
Ghanbari et al. [79]		1	0	1	0	0	0	0	0	1	1	4/10
Romero Morales et al. [80]		1	0	1	0	0	0	0	0	1	1	4/10
Moraska et al. [81]		1	0	1	0	0	0	1	0	1	1	5/10
Andersen et al. [82]		1	0	1	0	0	0	0	1	1	1	5/10

Criteria are available in https://pedro.org.au/english/resources/pedro-scale/ (accessed on 11 January 2022).

**Table 2:** Study characteristics.

Study	Sample Size	Population	Intervention Group	Comparison Group	Main outcomes and Measurement Tools	Follow-Up Period
Castien et al. [83]	80	Men and women aged 20–63 diagnosed with chronic TTH	Manual therapy treatment: mobilizations of the cervical and thoracic spine, postural correction and cervical stabilization exercises. 2 sessions/week, 9 sessions, 30 min/session.	No intervention	Intensity of pain: Numeric Rating Scale Headache frequency: personal diary headache duration personal diary	8 weeks 26 weeks
Ajimsha [84]	56	Men and women aged 18–50 diagnosed with TTH	TG1: Direct techniques for myofascial release in trapezius major, suboccipital, temporalis, neck flexors, mastoids muscles and Epicranial fascia. 2 sessions/week, 60 min/session. 12 weeks. TG2: Indirect techniques for myofascial release in skull base, posterior cervical musculature, hair, ears and facial musculature. 2 sessions/week, 60 min/session. 12 weeks.	No intervention	Headache frequency: personal diary	3 months
Da Silva et al. [85]	43	Pregnant women aged 18–39 diagnosed with TTH	Conventional acupuncture and additional points (trigger or painful points of splenius capitis, masseters and temporalis muscles) leaving the needles for 25 min. 2 session/week. 4 weeks	No intervention	Pain intensity (morning and evening): Numerical Rating Scale	3 months
Berggreen et al. [86]	38	Women aged 18–65 diagnosed with chronic TTH	Trigger Points treatment with frictions across or circulating in muscle fibers, ischemic compression, held from 30 to 60 s. Included muscles: trapezius (upper part), sternocleidomastoid, masseter, temporalis, medial and lateral pterygoid, anterior neck, facial, occipitofrontalis, splenius capitis and splenius cervicis, posterior cervical, and suboccipital muscles. 1 session/week. 10 weeks	No intervention	Pain intensity: Visual Analogical Scale	Pre-Post treatment
Espí-López et al. [87]	86	Men and women aged 18–65 diagnosed with TTH	TG1: Suboccipital soft tissue inhibition. 4 session/week. 4 weeks TG2: Occiput-atlas-axis manipulative treatment 4 session/week. 4 weeks TG3: G1 + G2 treatments combined. 4 session/week. 4 weeks.	Intragroup comparison	Pain Severity and headache frequency: Headache Disability Inventory	Pre-Post treatment
Moraska et al. [88]	62	Men and women aged 18–59 diagnosed with TTH	Massage: 15 min of myofascial of back, shoulders, chest, and neck; 20 min of trigger point release applied bilaterally to upper trapezius, suboccipital muscles, and both sternocleidomastoid; the final 10 min consisted of post-isometric relaxation directed at the right and left lateral cervical flexion, circular, or cross-fiber friction on the masseter, temporalis, and occipital-frontalis muscles, as well as gentle effleurage and petrissage on the neck and shoulders. 2 sessions/weeks. 6 weeks	Intragroup comparison	Pain intensity: Visual Analogical Scale. Frequency (episodes/week) Interview. Duration (hour/episode): Interview.	Pre-Post treatment and 1 month
Chassot et al. [89]	34	Women Aged 18–60 diagnosed with chronic TTH	Electroacupuncture: 16 acupuncture needles connected to an electro-stimulator for 30 min. 10 sessions. Twice/week. 5 weeks	Placebo	Pain intensity: visual analogical scale	Post treatment
Espí-López et al. [90]	102	Men and women aged 18–65 diagnosed with TTH	Occiput-atlas-axis manipulation (OAA) and soft tissue massage.1 session/week. 4 weeks	Massage 1 session/week. 4 weeks	Pain Severity and headache frequency: Headache Disability Inventory	Pre- Post treatment and 2 months
Ferragut-Garcías et al. [91]	97	Men and women aged 18–65 diagnosed with TTH	TG1: Soft tissues techniques.1 session/week.4 weeks TG2: Neural mobilization techniques 1 session/week.4 weeks TG3: Combined treatment involving soft tissue and neural mobilization techniques 1 session/week. 4 weeks	Placebo	Pain intensity: Visual analogical scale Headache frequency: personal diary	Post treatment and 1 month
Georgoudis et al. [92]	44	Men and women aged 18–65 diagnosed with TTH	Microwaves and myofascial release combined with acupuncture. 10 sessions.	Acupuncture	Pain intensity: Visual analogical scale	Pre-Post treatment
Pérez-Llanes et al. [93]	25	Men and women diagnosed with chronic TTH	Combined treatment with suboccipital muscle inhibition and interferential current. 2 sessions/week, 20 min/session.	Standard routine care (no interventions)	Intensity of pain: Numeric Rating Scale	4 weeks
Schiller et al. [94]	96	Men and women aged 18–65 diagnosed with chronic TTH	TG1: Acupuncture TG2: Medical training therapy (cardiovascular, strength-endurance, coordinative, proprioceptive, mobility and flexibility training), TG3: Combination of acupuncture and medical training. 6 weeks, 12 treatment units in decreasing frequency (3 sessions in weeks 1 and 2, 2 sessions in weeks 3 and 4, 1 session in weeks 5 and 6).	Standard routine care (no interventions)	Pain intensity: Verbal Rating Scale Frequency of headache: personal diary (days/month) Duration of headache: personal diary (hours/month) Frequency of headache medication intake: personal diary(days/month)	3 months 6 months
Corum et al. [95]	45	Men and women aged 19–48 diagnosed with TTH	TG1: Manipulation group: High-velocity low-amplitude (HVLA) manipulation plus exercise. 2 sessions/week for 4 weeks. TG2: Myofascial release group: suboccipital inhibition plus exercise. 2 sessions/week for 4 weeks.	Exercise intervention	Headache frequency: personal diary (days/2-week) Headache pain severity: Visual Analogical Scale	Pre-Post- treatment and 3 months
Gopichandran et al. [96]	169	Adult men and women diagnosed with chronic TTH	Progressive muscle relaxation (PMR) and deep breathing exercises. 12 weeks, 20 min,5 sessions/week, in the evening with an instruction booklet	Standard routine care (no interventions)	Pain intensity: Wong-Baker Faces Pain Scale	4 weeks 8 weeks 12 weeks
Runa et al. [97]	252	Men and women aged 18–75 diagnosed with anxiety and TTH	Warming acupuncture and moxibustion at temples combined with Deanxit (drug to improve depression and anxiety)	Warm acupuncture therapy (predominantly around the temple area)	Pain intensity: Visual Analogue Score Frequency of tension type headache: Interview Duration of tension type headache: Interview	Pre-Post treatment

TTH = Tension Type Headache, TG = Treatment Group.

**Table 3:** Results of individual studies.

Study	Intervention Group	Main Outcomes	Measurements Tools	Follow-Up Period	Effect Size (Cohen’sd)	95% CI (Cohen’s d)	*p* Values
Castien et al. [83]	Mobilizations of the cervical and thoracic spine, postural correction and cervical stabilization	Intensity of pain	Numeric Rating Scale	8 weeks	0.9	0.53 to 1.46	*p* = 0.003
				26 weeks	0.53	0.08 to 0.97	*p* = 0.027
		Headache frequency	Personal diary (14 days)	8 weeks	1.58	1.07 to 2.08	*p* < 0.001
				26 weeks	1.16	0.69 to 1.64	*p* < 0.001
		Headache duration	Personal diary 14 day (h/day)	8 weeks	0.56	0.12 to 1.01	*p* = 0.013
				26 weeks	0.39	−0.05 to 0.8	*p* = 0.095
Ajimsha et al. [84]	Direct techniques for myofascial release	Headache frequency	Personal diary	3 months	2.59	1.65 to 3.52	*p* < 0.001
	Indirect techniques for myofascial release	Headache frequency	Personal diary	3 months	3.4	2.35 to 4.5	*p* < 0.001
Da Silva et al. [85]	Conventional acupuncture and additional points	Pain intensity	Numerical Rating Scale	3 months	0.55	−0.05 to 1.16	*p* = 0.035
Berggreen et al. [86]	Trigger points treatment	Pain intensity (morning)	Visual Analogical Scale	Post treatment	0.66	0.014 to 1.32	*p* = 0.047
		Pain intensity (evening)	Visual Analogical Scale	Post treatment	0.17	−0.46 to 0.81	*p* = 0.594
Espí-López et al. [87]	Suboccipital soft tissue inhibition	Pain severity	Headache Disability Inventory.	Post treatment	0.31	−0.35 to 0.93	*p* = 0.04
		Headache frequency	Headache Disability Inventory	Post treatment	0.26	−0.37 to 0.91	*p* = 0.36
	Occiput-atlas-axis manipulative treatment	Pain severity	Headache Disability Inventory.	Post treatment	1.04	0.35 to 1.71	*p* = 0.004
		Headache frequency	Headache Disability Inventory.	Post treatment	0.59	−0.08 to 1.21	*p* = 0.03
	Combined treatment of both	Pain severity	Headache Disability Inventory.	Post treatment	0.80	0.067 to 1.38	*p* = 0.01
		Headache frequency	Headache Disability Inventory.	Post treatment	0.50	−0.012 to 1.18	*p* = 0.02
Moraska et al. [88]	Massage and trigger point treatment = 0.026	Pain intensity	Visual Analogical Scale	1 month	0.37	−0.28 to 1.03	*p* = 0.30
		Headache frequency	Interview (episodes/week)	1 month	0.54	−0.13 to 1.21	*p* = 0.026
		Headache duration	Interview (hour/episode)	1 month	0.32	−0.34 to 0.98	*p* = 0.49
Chassot et al. [89]	Electroacupuncture	Pain intensity	Visual Analogical Scale	Post treatment	0.63	0.05 to 1.32	*p* = 0.005
Espí-López et al. [90]	Occiput-atlas-axis manipulation and soft tissue massage	Pain severity	Headache Disability Inventory	Post treatment	0.38	0.0 to 0.77	*p* < 0.10
				2 months	0.28	−0.102 to 0.68	*p* = 0.31
		Headache frequency	Headache Disability Inventory.	Post treatment	0.35	0.03 to 0.75	*p* < 0.10
				2 months	0.63	0.23 to 1.03	*p* < 0.01
Ferragut-Garcías et al. [91]	Soft tissues techniques	Pain intensity	Visual Analogical Scale	Post treatment	2.35	1.61 to 3.09	*p* < 0.001
				1 month	2.47	1.71 to 3.23	*p* < 0.001
		Headache frequency	Personal diary	Post treatment	0.93	0.32 to 1.53	*p* < 0.001
				1 month	0.98	0.38 to 1.59	*p* < 0.001
	Neural mobilization techniques	Pain intensity	Visual Analogical Scale	Post treatment	1.27	0.65 to 1.88	*p* < 0.001
				1 month	1.29	0.68 to1.91	*p* < 0.001
		Headache frequency	Personal diary	Post treatment	1.17	0.57 to 1.78	*p* < 0.001
				1 month	1.11	0.51 to 1.71	*p* < 0.001
	Combined treatment of both	Pain intensity	Visual Analogical Scale	Post treatment	2.26	1.54 to 2.98	*p* < 0.001
				1 month	2.18	1.47 to 2.89	*p* < 0.001
		Headache frequency	Personal diary	Post treatment	1.50	0.86 to2.14	*p* < 0.001
				1 month	1.61	0.97 to 2.26	*p* < 0.001
Georgoudis et al. [92]	Microwaves and myofascial release combined with acupuncture	Pain intensity	Visual Analogical Scale	Post treatment	0.61	0.01 to 1.21	*p* < 0.05
Pérez-Llanes et al. [93]	Combined treatment with suboccipital muscle inhibition and interferential current	Intensity of pain	Numeric Rating Scale	4 weeks	1.13	-	*p* = 0.18
Schiller et al. [94] (*)	Acupuncture	Pain intensity	Verbal Rating Scale	3 months	0.83	-	*p* = 0.24
				6 months	0.44	-	-
		Frequency of headache	Personal diary (days/month)	3 months	0.14	-	-
				6 months	0.33	-	*p* = 0.01
		Duration of headache	Personal diary (hours/month)	3 months	0.14	-	-
				6 months	0.14	-	-
	Medical training therapy	Pain intensity	Verbal Rating Scale	3 months	0.33	-	*p* = 0.67
				6 months	0.88	-	-
		Frequency of headache	Personal diary (days/month)	3 months	0.69	-	-
				6 months	0.14	-	*p* = 0.01
		Duration of headache	Personal diary (hours/month)	3 months	0.25	-	-
				6 months	0.01	-	-
	Combination of acupuncture and medical training	Pain intensity	Verbal Rating Scale	3 months	1.25	-	*p* = 0.012
				6 months	0.29	-	-
		Frequency of headache	Personal diary (days/month)	3 months	0.37	-	-
				6 months	0.03	-	*p* = 0.04
		Duration of headache	Personal diary (hours/month)	3 months	0.05	-	-
				6 months	0.34	-	-
Corum et al. [95]	High-velocity low-amplitude (HVLA) manipulation	Headache frequency	Personal diary (days/2-week)	Post treatment	1.58	-	*p* < 0.05
				3 months	1.32	-	*p* < 0.05
		Pain intensity	Visual Analogue Scale	Post treatment	2.77	-	*p* < 0.05
				3 months	1.99	-	*p* < 0.05
	Suboccipital inhibition	Headache frequency	Personal diary (days/2-week)	Post treatment	0.64	-	*p* < 0.05
				3 months	0.48	-	*p* < 0.05
		Pain intensity	Visual Analogue Scale	Post treatment	1.07	-	*p* < 0.05
				3 months	0.6	-	*p* < 0.05
Gopichandran et al. [96]	Progressive muscle relaxation (PMR) and deep breathing exercises	Pain intensity	Wong-Baker Faces Pain Scale	4 weeks	1.7	-	*p* < 0.001
				8 weeks	2.27	-	*p* < 0.001
				12 weeks	2.99	-	*p* < 0.001
Runa et al. [97]	Moxibustion at temples combined with Deanxit	Pain intensity	Visual Analogue Score	Post treatment	8.35	-	*p* < 0.05
		Frequency of tension type headache	Interview	Post treatment	1.81	-	*p* < 0.05
		Duration of tension type headache	Interview	Post treatment	2.83	-	*p* < 0.05

(*) Data after 3 and 6 months, comparing them with control group.

## Data Availability

Not applicable.

## References

[1] De Tommaso M., Fernández-de-las-Peñas C. (2016). Tension Type Headache. Curr. Rheumatol. Rev..

[2] Stovner L.J., Hagen K., Linde M., Steiner T.J. (2022). The global prevalence of headache: An update, with analysis of the influences of methodological factors on prevalence estimates. J. Headache Pain.

[3] Olesen J. (2018). Headache Classification Committee of the International Headache Society (IHS) The International Classification of Headache Disorders, 3rd edition. Cephalalgia.

[4] Freitag F. (2013). Managing and Treating Tension-type Headache. Med. Clin. N. Am..

[5] Jensen R.H. (2018). Tension-Type Headache—The Normal and Most Prevalent Headache. Headache.

[6] Bendtsen L., Ashina S., Moore A., Steiner T.J. (2015). Muscles and their role in episodic tension-type headache: Implications for treatment. Eur. J. Pain.

[7] Rains J.C., Davis R.E., Smitherman T.A. (2015). Tension-Type Headache and Sleep. Curr. Neurol. Neurosci. Rep..

[8] Haque B., Rahman K.M., Hoque A., Hasan A.H., Chowdhury R.N., Khan S.U., Alam M.B., Habib M., Mohammad Q.D. (2012). Precipitating and relieving factors of migraine versus tension type headache. BMC Neurol..

[9] Safiri S., Kolahi A.-A., Noori M., Nejadghaderi S.A., Aslani A., Sullman M.J.M., Farhoudi M., Araj-Khodaei M., Collins G.S., Kaufman J.S. (2022). Burden of tension-type headache in the Middle East and North Africa region, 1990–2019. J. Headache Pain.

[10] Cigarán-Méndez M., Jiménez-Antona C., Parás-Bravo P., Fuensalida-Novo S., Rodríguez-Jiménez J., Fernández-De-Las-Peñas C. (2019). Active Trigger Points Are Associated with Anxiety and Widespread Pressure Pain Sensitivity in Women, but not Men, With Tension Type Headache. Pain Pract..

[11] Fernández-De-Las-Peñas C., Arendt-Nielsen L. (2017). Improving understanding of trigger points and widespread pressure pain sensitivity in tension-type headache patients: Clinical implications. Expert Rev. Neurother..

[12] Moher D., Liberati A., Tetzlaff J., Altman D.G., The PRISMA Group (2009). Preferred reporting items for systematic reviews and meta-analyses: The PRISMA statement. Ann. Intern. Med..

[13] Maher C.G., Sherrington C., Herbert R.D., Moseley A.M., Elkins M. (2003). Reliability of the PEDro Scale for Rating Quality of Randomized Controlled Trials. Phys. Ther..

[14] Sun-Edelstein C., Mauskop A. (2012). Complementary and alternative approaches to the treatment of tension-type headache. Curr. Pain Headache Rep..

[15] Espí-López G., Arnal-Gómez A., Arbós-Berenguer T., López González Á.A., Vicente-Herrero T. (2014). Effectiveness of Physical Therapy in Patients with Tension-type Headache: Literature Review. J. Jpn. Phys. Ther. Assoc..

[16] Chaibi A., Russell M.B. (2014). Manual therapies for primary chronic headaches: A systematic review of randomized controlled trials. J. Headache Pain.

[17] Nicholson R.A., Buse D.C., Andrasik F., Lipton R.B. (2011). Nonpharmacologic Treatments for Migraine and Tension-Type Headache: How to Choose and When to Use. Curr. Treat. Options Neurol..

[18] Monticco A., Granato A., Menegoni M., Deodato M., Fantini J., Marcovich R. (2015). O031. Physiotherapy treatment in chronic tension-type headache: An ongoing study. J. Headache Pain.

[19] Castien R., Blankenstein A., De Hertogh W. (2015). Pressure pain and isometric strength of neck flexors are related in chronic tension-type headache. Pain Physician.

[20] Castien R., Blankenstein A., van der Windt D., Heymans M.W., Dekker J. (2013). The Working Mechanism of Manual Therapy in Participants with Chronic Tension-Type Headache. J. Orthop. Sports Phys. Ther..

[21] Gil-Martínez A., Kindelan-Calvo P., Agudo-Carmona D., Muñoz-Plata R., López-De-Uralde-Villanueva I., La Touche R. (2013). Therapeutic exercise as treatment for migraine and tension-type headaches: A systematic review of randomised clinical trials. Rev. Neurol..

[22] Diazgranados Sánchez J.A., Chan Guevara L.S., Valencia Artunduaga M.H., Piedrahita P.A., Echeverry A.F., Ramos Burbano G.E. (2015). Cefalea crónica tipo tensión: Una nueva experiencia de tratamiento Chronictension-typeheadache: A new treatmentexperience Trabajo original. Acta Neurol. Colomb..

[23] Milka D., Jachacz-Lopata M., Kmita B., Likus W., Bajor G. Efficacy of manual therapy techniques in tension-type headache. Proceedings of the 7th World Congress of the World Institute of Pain, WIP 2014.

[24] Fricton J.R., Ouyang W., Nixdorf D.R., Schiffman E.L., Velly A.M., Look J.O. (2010). Critical appraisal of methods used in randomized controlled trials of treatments for temporomandibular disorders. J. Orofac. Pain.

[25] Marchand A.A., Cantin V., Murphy B., Stern P., Descarreaux M. (2014). Is performance in goal oriented head movements altered in patients with tension type headache?. BMC Musculoskelet. Disord..

[26] Domingues R.B., Duarte H., Rocha N.P., Teixeira A.L. (2015). Neurotrophic factors in tension-type headache. Arq. Neuro-Psiquiatr..

[27] Sertel M., Bakar Y., Şimşek T.T. (2017). The effect of body awareness therapy and aerobic exercises on pain and quality of life in the patients with tension type headache. Afr. J. Tradit. Complement. Altern. Med..

[28] Tabeeva G.R., Fokina N.M. (2016). Possibilities of preventive therapy in frequent episodic tension-type headache. Zhurnal Nevrol. Psikhiatrii Im. SS Korsakova.

[29] lvarez-Melcón A.C., Valero-Alcaide R., Atín-Arratibel M.A., Melcón-Álvarez A., Beneit-Montesinos J.V. (2018). Effects of physical therapy and relaxation techniques on the parameters of pain in university students with tension-type headache: A randomised controlled clinical trial. Neurologia.

[30] Castien R., de Hertogh W. (2019). A Neuroscience Perspective of Physical Treatment of Headache and Neck Pain. Front. Neurol..

[31] Chatchawan U., Thongbuang S., Yamauchi J. (2019). Characteristics and distributions of myofascial trigger points in individuals with chronic tension-type headaches. J. Phys. Ther. Sci..

[32] Qu P., Yu J.-X., Xia L., Chen G.-H. (2018). Cognitive Performance and the Alteration of Neuroendocrine Hormones in Chronic Tension-Type Headache. Pain Pract..

[33] Speciali J.G., Dach F. (2015). Temporomandibular Dysfunction and Headache Disorder. Headache.

[34] Kim J., Cho S.-J., Kim W.-J., Yang K.I., Yun C.-H., Chu M.K. (2017). Insomnia in tension-type headache: A population-based study. J. Headache Pain.

[35] Deodato M., Guolo F., Monticco A., Fornari M., Manganotti P., Granato A. (2019). Osteopathic Manipulative Therapy in Patients with Chronic Tension-Type Headache: A Pilot Study. J. Am. Osteopat. Assoc..

[36] Falsiroli Maistrello L., Geri T., Gianola S., Zaninetti M., Testa M. (2018). Effectiveness of Trigger Point Manual Treatment on the Frequency, Intensity, and Duration of Attacks in Primary Headaches: A Systematic Review and Meta-Analysis of Randomized Controlled Trials. Front. Neurol..

[37] Garrigós-Pedrón M., La Touche R., Navarro-Desentre P., Gracia-Naya M., Segura-Ortí E. (2018). Effects of a Physical Therapy Protocol in Patients with Chronic Migraine and Temporomandibular Disorders: A Randomized, Single-Blinded, Clinical Trial. J. Oral Facial Pain Headache.

[38] Gildir S., Tüzün E.H., Eroğlu G., Eker L. (2019). A randomized trial of trigger point dry needling versus sham needling for chronic tension-type headache. Medicine.

[39] Irimia P., Martínez-Vila E., Irimia P., Martínez-Vila E. (2019). Fisioterapia en cefalea tensional. ¿Debe recomendarse a nuestros pacientes?. An. Sist. Sanit. Navar..

[40] Jiang W., Li Z., Wei N., Chang W., Chen W., Sui H.J. (2019). Effectiveness of physical therapy on the suboccipital area of patients with tension-type headache: A meta-analysis of randomized controlled trials. Medicine.

[41] Kamali F., Mohamadi M., Fakheri L., Mohammadnejad F. (2019). Dry needling versus friction massage to treat tension type headache: A randomized clinical trial. J. Bodyw. Mov. Ther..

[42] Maistrello L.F., Rafanelli M., Turolla A. (2019). Manual Therapy and Quality of Life in People with Headache: Systematic Review and Meta-analysis of Randomized Controlled Trials. Curr. Pain Headache Rep..

[43] Mohamed A.A., Shendy W.S., Semary M., Mourad H.S., Battecha K.H., Soliman E.S., EL Sayed S.H., Mohamed G.I. (2019). Combined use of cervical headache snag and cervical snag half rotation techniques in the treatment of cervicogenic headache. J. Phys. Ther. Sci..

[44] Pourahmadi M., Mohseni-Bandpei M.A., Keshtkar A., Koes B.W., Fernández-De-Las-Peñas C., Dommerholt J., Bahramian M. (2019). Effectiveness of dry needling for improving pain and disability in adults with tension-type, cervicogenic, or migraine headaches: Protocol for a systematic review. Chiropr. Man. Ther..

[45] Saha F.J., Pulla A., Ostermann T., Miller T., Dobos G., Cramer H. (2019). Effects of occlusal splint therapy in patients with migraine or tension-type headache and comorbid temporomandibular disorder: A randomized controlled trial. Medicine.

[46] Castien R.F., van der Windt D.A., Blankenstein A.H., Heymans M.W., Dekker J. (2012). Clinical variables associated with recovery in patients with chronic tension-type headache after treatment with manual therapy. Pain.

[47] Luedtke K., Allers A., Schulte L.H., May A. (2016). Efficacy of interventions used by physiotherapists for patients with headache and migraine—Systematic review and meta-analysis. Cephalalgia.

[48] Rolle G., Tremolizzo L., Somalvico F., Ferrarese C., Bressan L.C. (2014). Pilot Trial of Osteopathic Manipulative Therapy for Patients with Frequent Episodic Tension-Type Headache. J. Am. Osteopat. Assoc..

[49] Gui-Demase M.S., Silva K.C.D., Teixeira G.D.S. (2021). La terapia manual asociada con calor superficial redujo el dolor y la automedicación en pacientes con cefalea tensional. Fisioter. Pesqui..

[50] Elchami Z., Issa M.B., Mohamadin A.S.A., Massoud R., Magayano D. (2015). The effectiveness of CoQ10 in the treatment of Tension-Type Headache. Cephalalgia.

[51] Chatchawan U., Eungpinichpong W., Sooktho S., Tiamkao S., Yamauchi J. (2014). Effects of Thai Traditional Massage on Pressure Pain Threshold and Headache Intensity in Patients with Chronic Tension-Type and Migraine Headaches. J. Altern. Complement. Med..

[52] Duymaz T. (2021). Efficacy of kinesio taping on pain, pain threshold and emotional status in tension-type headache. Gazz. Med. Ital. Arch. Sci. Med..

[53] Bono F., Salvino D., Mazza M.R., Curcio M., Trimboli M., Vescio B., Quattrone A. (2015). The influence of ictal cutaneous allodynia on the response to occipital transcutaneous electrical stimulation in chronic migraine and chronic tension-type headache: A randomized, sham-controlled study. Cephalalgia.

[54] Hirsvang J., Kroell L., Caspersen N., Madsen B., Jensen R. (2014). EHMTI-0159. Assessment of functional health and well-being in headache patients: The effect of individual-based physical therapy. J. Headache Pain.

[55] Mongini F., Evangelista A., Milani C., Ferrero L., Ciccone G., Ugolini A., Piedimonte A., Sigaudo M., Carlino E., Banzatti E. (2012). An Educational and Physical Program to Reduce Headache, Neck/Shoulder Pain in a Working Community: A Cluster-Randomized Controlled Trial. PLoS ONE.

[56] Bembalgi V., Naik K.R. (2012). Galvanic skin resistance (GSR) biofeedback in tension-type headache—Auditory, visual or combined feedback: Which is beneficial? A randomized controlled trial. Adv. Physiother..

[57] Cachinero-Torre A., Díaz-Pulido B., del Barco A.A. (2017). Relationship of the Lateral Rectus Muscle, the Supraorbital Nerve, and Binocular Coordination with Episodic Tension-Type Headaches Frequently Associated with Visual Effort. Pain Med..

[58] López G.V.E., Rodríguez-Blanco C., Oliva-Pascual-Vaca Á., Molina-Martinez F.J., Falla D. (2016). Do manual therapy techniques have a positive effect on quality of life in people with tension-type headache? A randomized controlled trial. Eur. J. Phys. Rehabil. Med..

[59] Rota E., Evangelista A., Ceccarelli M., Ferrero L., Milani C., Ugolini A., Mongini F. (2016). Efficacy of a workplace relaxation exercise program on muscle tenderness in a working community with headache and neck pain: A longitudinal, controlled study. Eur. J. Phys. Rehabil. Med..

[60] Choi S.-Y., Choi J.-H. (2016). The effects of cervical traction, cranial rhythmic impulse, and Mckenzie exercise on headache and cervical muscle stiffness in episodic tension-type headache patients. J. Phys. Ther. Sci..

[61] Bakhshani N.M., Amirani A., Amirifard H., Shahrakipoor M. (2015). The Effectiveness of Mindfulness-Based Stress Reduction on Perceived Pain Intensity and Quality of Life in Patients with Chronic Headache. Glob. J. Health Sci..

[62] Al-Hashel J.Y., Ahmed S.F., AlShawaf F.J., Alroughani R. (2018). Use of traditional medicine for primary headache disorders in Kuwait. J. Headache Pain.

[63] Georgoudis G., Felah B., Nikolaidis P.T., Papandreou M., Mitsiokappa E., Mavrogenis A.F., Rosemann T., Knechtle B. (2018). The effect of physiotherapy and acupuncture on psychocognitive, somatic, quality of life, and disability characteristics in TTH patients. J. Pain Res..

[64] Krøll L.S., Hammarlund C.S., Linde M., Gard G., Jensen R.H. (2018). The effects of aerobic exercise for persons with migraine and co-existing tension-type headache and neck pain. A randomized, controlled, clinical trial. Cephalalgia.

[65] Bodes-Pardo G., Pecos-Martin D., Izquierdo T.G., Salom-Moreno J., Fernández-De-Las-Peñas C., Ortega-Santiago R. (2013). Manual Treatment for Cervicogenic Headache and Active Trigger Point in the Sternocleidomastoid Muscle: A Pilot Randomized Clinical Trial. J. Manip. Physiol. Ther..

[66] Langdon R., Taraman S. (2018). Posttraumatic Headache. Pediatr. Ann..

[67] Lindfors E., Magnusson T., Ernberg M. (2020). Effect of Therapeutic Jaw Exercises in the Treatment of Masticatory Myofascial Pain: A Randomized Controlled Study. J. Oral Facial Pain Headache.

[68] Moseley A.M., Herbert R., Sherrington C., Maher C.G. (2002). Evidence for physiotherapy practice: A survey of the Physiotherapy Evidence Database (PEDro). Aust. J. Physiother..

[69] Söderberg E.I., Carlsson J.Y., Stener-Victorin E., Dahlöf C. (2011). Subjective well-being in patients with chronic tension-type headache: Effect of acupuncture, physical training, and relaxation training. Clin. J. Pain.

[70] Monzani L., Espí-López G., Zurriaga R., Andersen L.L. (2016). Manual therapy for tension-type headache related to quality of work life and work presenteeism: Secondary analysis of a randomized controlled trial. Complement. Ther. Med..

[71] Azam M.A., Katz J., Mohabir V., Ritvo P. (2016). Individuals with tension and migraine headaches exhibit increased heart rate variability during post-stress mindfulness meditation practice but a decrease during a post-stress control condition—A randomized, controlled experiment. Int. J. Psychophysiol..

[72] Vernon H., Borody C., Harris G., Muir B., Goldin J., Dinulos M. (2015). A Randomized Pragmatic Clinical Trial of Chiropractic Care for Headaches with and Without a Self-Acupressure Pillow. J. Manip. Physiol. Ther..

[73] del-Blanco-Muñiz J.Á., Laguarta-Val S., Fernández de-Las-Peñas C. (2018). Evaluación y mejora de la calidad asistencial en fisioterapia a pacientes con cefalea. An. Sist. Sanit. Navar..

[74] Madsen B.K., Søgaard K., Andersen L.L., Tornøe B., Jensen R.H. (2018). Efficacy of strength training on tension-type headache: A randomised controlled study. Cephalalgia.

[75] Mahmoud Sawan S.A., Abass A., Alkharbotl A.M. (2021). Laser acupuncture for relieve peri-cranial tenderness with tension-type headache patients: A randomized control trial. Fizjoterapia Pol..

[76] Mohamadi M., Rojhani-Shirazi Z., Assadsangabi R., Rahimi-Jaberi A. (2020). Can the Positional Release Technique Affect Central Sensitization in Patients with Chronic Tension-Type Headache? A Randomized Clinical Trial. Arch. Phys. Med. Rehabil..

[77] Choi W. (2021). Effect of 4 Weeks of Cervical Deep Muscle Flexion Exercise on Headache and Sleep Disorder in Patients with Tension Headache and Forward Head Posture. Int. J. Environ. Res. Public Health.

[78] Espí-López G.V., Gómez-Conesa A., Gómez A.A., Martínez J.B., Pascual-Vaca Á.O., Blanco C.R. (2014). Treatment of tension-type headache with articulatory and suboccipital soft tissue therapy: A double-blind, randomized, placebo-controlled clinical trial. J. Bodyw. Mov. Ther..

[79] Ghanbari A., Rahimijaberi A., Mohamadi M., Abbasi L., Sarvestani F.K. (2012). The effect of trigger point management by positional release therapy on tension type headache. Neurorehabilitation.

[80] Romero Morales C., Cabrera Guerra M., Gómez Ruano M.A., Jiménez Saiz S. (2015). Effectiveness of cervical manipulation vs. positional release therapy in trigger points for tension type headache. Fisioterapia.

[81] Moraska A.F., Schmiege S.J., Mann J.D., Butryn N., Krutsch J.P. (2017). Responsiveness of Myofascial Trigger Points to Single and Multiple Trigger Point Release Massages: A Randomized, Placebo Controlled Trial. Am. J. Phys. Med. Rehabil..

[82] Andersen C.H., Jensen R.H., Dalager T., Zebis M.K., Sjøgaard G., Andersen L.L. (2017). Effect of resistance training on headache symptoms in adults: Secondary analysis of a RCT. Musculoskelet. Sci. Pract..

[83] Castien R.F., van der Windt D.A., Grooten A., Dekker J. (2011). Effectiveness of manual therapy for chronic tension-type headache: A pragmatic, randomised, clinical trial. Cephalalgia.

[84] Ajimsha M.S. (2011). Effectiveness of direct vs indirect technique myofascial release in the management of tension-type headache. J. Bodyw. Mov. Ther..

[85] Da Silva J.B.G., Nakamura M.U., Cordeiro J.A., Kulay L. (2012). Acupuncture for tension-type headache in pregnancy: A prospective, randomized, controlled study. Eur. J. Integr. Med..

[86] Berggreen S., Wiik E., Lund H. (2012). Treatment of myofascial trigger points in female patients with chronic tension-type headache—A randomized controlled trial. Adv. Physiother..

[87] Espí-López G.V., Rodriguez-Blanco C., Pascual-Vaca Á.O., Benitez-Martinez J.C., Lluch E., Falla D. (2014). Effect of manual therapy techniques on headache disability in patients with tension-type headache. Randomized controlled trial. Eur. J. Phys. Rehabil. Med..

[88] Moraska A.F., Stenerson L., Butryn N., Krutsch J.P., Schmiege S.J., Mann J.D. (2015). Myofascial trigger point-focused head and neck massage for recurrent tension-type headache: A randomized, placebo-controlled clinical trial. Clin. J. Pain.

[89] Chassot M., Dussan-Sarria J.A., Sehn F.C., Deitos A., de Souza A., Vercelino R., Torres I.L., Fregni F., Caumo W. (2015). Electroacupuncture analgesia is associated with increased serum brain-derived neurotrophic factor in chronic tension-type headache: A randomized, sham controlled, crossover trial. BMC Complement. Altern. Med..

[90] López G.V.E., Zurriaga-Llorens R., Monzani L., Falla D. (2016). The effect of manipulation plus massage therapy versus massage therapy alone in people with tension-type headache. a randomized controlled clinical trial. Eur. J. Phys. Rehabil. Med..

[91] Ferragut-Garcías A., Plaza-Manzano G., Rodríguez-Blanco C., Velasco-Roldán O., Pecos-Martín D., Oliva-Pascual-Vaca J., Llabrés-Bennasar B., Oliva-Pascual-Vaca Á. (2017). Effectiveness of a Treatment Involving Soft Tissue Techniques and/or Neural Mobilization Techniques in the Management of Tension-Type Headache: A Randomized Controlled Trial. Arch Phys. Med. Rehabil..

[92] Georgoudis G., Felah B., Nikolaïdis P., Damigos D. (2017). The effect of myofascial release and microwave diathermy combined with acupuncture versus acupuncture therapy in tension-type headache patients: A pragmatic randomized controlled trial. Physiother. Res. Int..

[93] Pérez-Llanes R., Ruiz-Cárdenas J.D., Meroño-Gallut A.J., Fernández-Calero M.I., Ríos-Díaz J. (2020). Effectiveness of suboccipital muscle inhibition combined with interferential current in patients with chronic tension-type headache: A randomised controlled clinical trial. Neurologia.

[94] Schiller J., Karst M., Kellner T., Zheng W., Niederer D., Vogt L., Eckhardt I., Beissner F., Korallus C., Sturm C. (2021). Combination of acupuncture and medical training therapy on tension type headache: Results of a randomised controlled pilot study. Cephalalgia.

[95] Corum M., Aydin T., Ceylan C.M., Kesiktas F.N. (2021). The comparative effects of spinal manipulation, myofascial release and exercise in tension-type headache patients with neck pain: A randomized controlled trial. Complement. Ther. Clin. Pract..

[96] Gopichandran L., Srivastsava A.K., Vanamail P., Kanniammal C., Valli G., Mahendra J., Dhandapani M. (2021). Effectiveness of Progressive Muscle Relaxation and Deep Breathing Exercise on Pain, Disability, and Sleep Among Patients with Chronic Tension-Type Headache: A Randomized Control Trial. Holist. Nurs. Pract..

[97] Runa A., Bao Q., Sai Y.C., Te M., Hu R., Sa R., Mu R., Bo A. (2021). Clinical observation of warming acupuncture and moxibustion at the temples combined with Deanxit in the treatment of tension headache with anxiety and depression: A retrospective study. Ann. Palliat. Med..

[98] Krøll L.S., Callesen H.E., Carlsen L.N., Birkefoss K., Beier D., Christensen H.W., Jensen M., Tómasdóttir H., Würtzen H., Høst C.V. (2021). Manual joint mobilisation techniques, supervised physical activity, psychological treatment, acupuncture and patient education for patients with tension-type headache. A systematic review and meta-analysis. J. Headache Pain.

